# Older adults' physical activity after lockdown: Testing the health action process approach and the moderating role of fear of Covid‐19

**DOI:** 10.1111/aphw.12384

**Published:** 2022-07-12

**Authors:** Valérie D. Bösch, Jennifer Inauen

**Affiliations:** ^1^ Department of Health Psychology and Behavioral Medicine University of Bern Bern Switzerland

**Keywords:** Covid‐19, fear of Covid‐19, health action process approach, older adults, physical activity

## Abstract

The coronavirus pandemic has influenced many lives, particularly older adults'. Although isolation protects from infection, health behaviors like physical activity (PA) are important to reinstate after lockdown. However, fear of Covid‐19 may act as a barrier, for example, by preventing people from going outside. Based on the health action process approach (HAPA), we investigated whether and why older adults' PA changed after lockdown, and whether fear of Covid‐19 moderates the intention–behavior relationship. Participants of this longitudinal study aged 65+ from German‐speaking Europe completed an online questionnaire about their PA, fear of Covid‐19, and HAPA factors in April and May 2020. Data were analyzed using multiple linear regressions. Results showed that moderate to vigorous activity (MVPA) remained stable after lockdown and that self‐efficacy most robustly influenced the intention to be active. PA was not explained by any volitional factor but was strongly related to past PA. Interestingly, the relationship of past and future MVPA was attenuated by fear of Covid‐19, but this finding was not robust when outliers were removed. In conclusion, self‐efficacy is the most important motivator for PA in older adults after an interruption like a lockdown. Strong physical activity habits may facilitate PA after a period of isolation.

## BACKGROUND

Coronavirus Disease 2019 (Covid‐19) is a viral respiratory disease that has affected and will continue to affect millions of people worldwide (Moro & Paoli, 2020). Due to the rising numbers of infections, many countries implemented restrictions early in 2020 to slow the spread of the virus and thus to prevent health systems from collapse. Restrictions included lockdowns in many countries, in which a range of social interactions were prohibited, shops were shut, and stay‐at‐home policies were instated (Han et al., 2020). One group that was substantially affected by these governmental measures were older adults.

### Older adults during the Covid‐19 pandemic

Due to risks of complications and mortality that was potentially higher than for those to the general population, adults older than 65 years were declared a risk group and advised to stay at home (Shahid et al., 2020). Older adults' vulnerability to contracting Covid‐19 was extensively discussed in the media, leading to high avoidance and fear among this age group (Rahman & Bahar, 2020). However, even during such a time, maintaining health behaviors, such as regular physical activity, is important for physical and mental health (Inauen & Zhou, 2020).

Regardless of their health status, older adults can benefit from physical activity (Hupin et al., 2015). Physical activity has conferred multiple benefits particularly during the pandemic. First, physically active individuals have better control over high‐risk comorbidities, such as cardiovascular diseases and diabetes, which can increase susceptibility to severe complications from Covid‐19 (Moro & Paoli, 2020), engaging in moderate‐to‐vigorous physical activity is recommended (Bull et al., 2020; World Health Organization, 2020). However, even light physical activity during the Covid‐19 pandemic can help alleviate some of the negative mental health impacts that older adults may experience while isolated (Callow et al., 2020). Notably, maintaining a healthy life style can influence the perceived quality of life during the pandemic in older adults (Duan et al., 2021).

In spite of these benefits, activity tracker data suggest that step counts decreased worldwide after Covid‐19 was declared a global pandemic (Tison et al., 2020; Warren & Skillman, 2020). Moreover, there is evidence that older adults in particular engaged in less physical activity during the first wave of the pandemic (Rhodes et al., 2020). This is further substantiated by older adults' self‐reports of decreased physical activity (Visser et al., 2020). Liang et al. (2021) for example found that 35% of older adults reported a decrease in physical activity in the first wave of the pandemics. Also, the odds of decreased vigorous activity after lockdown is bigger in older adults than in other populations (Bu et al., 2021). This suggests that the Covid‐19 pandemic can negatively affect older adults' physical activity in the long term (Hall et al., 2021). To avoid such prolonged effects, recovering physical activity is important. However, it is unknown whether and which older adults recover their physical activity as Covid‐19‐related public health restrictions are lifted.

### The health action process approach (HAPA)—An explanatory model of physical activity

A model that can provide insight into the recovery of physical activity is the HAPA (Schwarzer, 2008). The HAPA model describes how motivational and volitional factors influence health behavior intentions and actions. It suggests a distinction between pre‐intentional motivation processes, collectively termed the “motivational phase,” which lead to a behavioral intention, and post‐intentional volition processes also termed the “volitional phase”, that lead to the actual health behavior. In the motivational phase, risk perception, outcome expectancies, and self‐efficacy predict the intention. Risk perception may involve, for instance, people perceiving the risk that lack of physical activity may lead to cardiovascular diseases (Dubbert et al., 2002; Schwarzer, 2008). In contrast, outcome expectancies are formed when people balance the consequences of certain behavioral outcomes. Thus, people may decide to go for a walk because they are sure that it boosts their well‐being while in isolation. Lastly, self‐efficacy means that an individual believes in their own capability to perform a desired action. For example, when leaving the house is not advised, an individual must be sure of their capabilities of remaining physically active at home, even if they do not have the same equipment as a gym. All three factors then predict the intention to perform a certain target behavior.

In the volitional phase, coping plans, action plans, and action control are crucial for the adoption, maintenance, and recovery of a behavior (Schwarzer, 2008). Coping and action plans mediate the effect of the intention on behavior. Coping plans are formed in the anticipation of barriers by generating alternative behaviors to overcome them (Schwarzer, 2008). For example, if someone cannot go to their fitness class due to Covid‐19 restrictions, they can instead identify activities that they can perform from home. Action plans, in turn, include specific situational cues (when and where to be active) that are linked to the desired action (Hagger & Luszczynska, 2014; Schwarzer, 2008). Adapting to the current pandemic, individuals may plan to go for a walk in the park in the evening, because such choices minimize the number of other people in their vicinity. Lastly, action control involves self‐regulatory processes that enable the maintenance of a behavior (Sniehotta et al., 2005). It describes the degree of control someone can exert despite internal or external factors that interfere with the execution of a behavior (Kuhl & Beckmann, 2012).

Empirical evidence overall strongly supports the assumptions of the HAPA model (Zhang et al., 2019), including for physical activity for various groups (Barg et al., 2012; Caudroit et al., 2011; Parschau et al., 2014). Therefore, the HAPA model can be considered a suitable theoretical framework for this study. However, previous studies that used the HAPA model have predominantly focused on the adoption and sometimes maintenance of physical activity. Less attention has been paid to the recovery of physical activity after an interruption, such as the first Covid‐19 lockdown.

### Recovering physical activity after an interruption: The role of fear of Covid‐19

Although evidence shows that many people reduced (and others increased) their physical activity during the first Covid‐19‐related lockdown (Naughton et al., 2020), little is known about whether physical activity recovered when lockdown restrictions were lifted, and if so whose. Besides the predictors of health behavior change such as those specified by the HAPA model, there is evidence that the fear of Covid‐19 could be an important explanatory factor.

Presti et al. (2020) define fear as an emotional reaction that occurs in the presence of a danger and is often accompanied by emotional distress and behavioral avoidance. The role of fear of Covid has been extensively examined with respect to preventive behaviors (e.g., Pakpour & Griffiths, 2020; Stolow et al., 2020). Fear of Covid‐19 was found to correlate significantly with such avoidant behaviors as staying at home (Jørgensen et al., 2020), which could disrupt regular physical activity. Moreover, the fear of Covid‐19 positively predicted public health compliance in the Covid‐19 pandemic (Harper et al., 2020). However, little to nothing is known about whether and how fear of Covid‐19 influences health behaviors. Previous research on positive emotions and health behavior has shown that emotions relate to health behavior by moderating the intention–behavior relationship. For example, positive affective responses like expected pleasure, enjoyment and exercise affect can have an effect on the translation of intentions into physical activity (Kwan & Bryan, 2010; Rhodes & Dickau, 2013). Negative emotions may similarly moderate the intention–behavior relationship, although likely in the opposite direction. Based on the concept of behavioral avoidance, fear of Covid‐19 may act as a behavioral barrier. Persons with greater fear of Covid‐19 may feel a stronger need to stay indoors to protect themselves from infection, which thus may inhibit the enactment of physical activity intentions.

#### Purpose of the present study

The aims of the present study are first to investigate the change in older adults' physical activity after the first Covid‐19 lockdown. Second, we investigate whether the HAPA model can explain the intention to be active and physical activity after the restrictions were lifted. Third, we investigate whether fear of Covid‐19 acts as a barrier to physical activity after lockdown. We hypothesized that the higher risk perception, positive outcome expectancies, and self‐efficacy are, the higher is the intention to be physically active after lockdown. For the action model, we also hypothesized that the higher the intention and action control and the more detailed an older adult's action and coping plans are, the more physically active they will be after lockdown. Lastly, we hypothesized that fear of Covid‐19 moderates the relationship between intention and physical activity after lockdown by inhibiting the translation of intentions into action in fearful individuals.

## METHODS

This study was part of a larger 3‐wave panel study that took place between April and August 2020. The present study analyzed the first two time points (T1 and T2). These time points were the closest to the lockdown, making this a suitable time window for investigating the recovery of the intention to be active and physical activity after lockdown. Additionally, high attrition at T3 impeded conducting a multiwave analysis.

**FIGURE 1:**
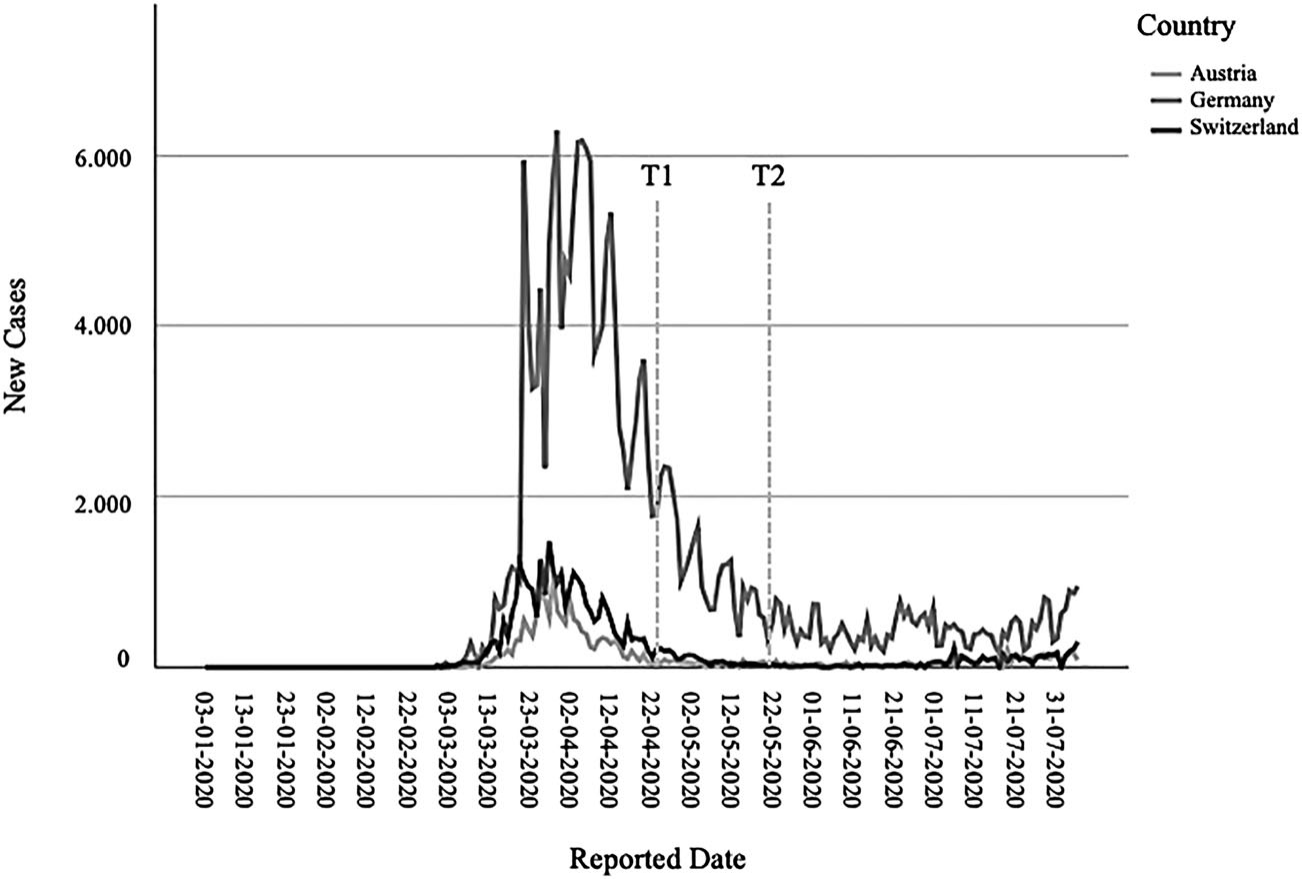
Survey waves plotted against the reported Covid‐19 cases for the first wave of the pandemic in Austria, Germany, and Switzerland. Note. T1 to T3 = survey waves. Data retrieved from https://covid19.who.int/WHO‐COVID‐19‐global‐data.csv

T1 data collection started on April 21, 2020, approximately 1 month after the initiation of the lockdown, which started in Austria on March 18, Switzerland on March 19, and Germany on March 23, 2020 (see Plümper & Neumayer, 2022). T2 started on May 21. See Figure 1 for an overview of the data collection contrasted on the course of the first pandemic wave. The Ethics Committee of the University of Bern (Nr. 2020‐04‐00012) approved this study.

### Procedures

The sample size was estimated via a priori power analysis using the software package, GPower (Faul & Erdfelder, 1992). The sample size of 395 was estimated for finding a small effect (*f*_*2*_ = .02) for a significance level of *α* = .05 and a power of .80.

Participants (*N* = 263; targeted *N* = 395) were recruited Germany, Switzerland, and Austria. To take part in the study, participants had to be at least 65 years old, speak German, and be willing to be recontacted for the subsequent panels of the survey.

**FIGURE 2:**
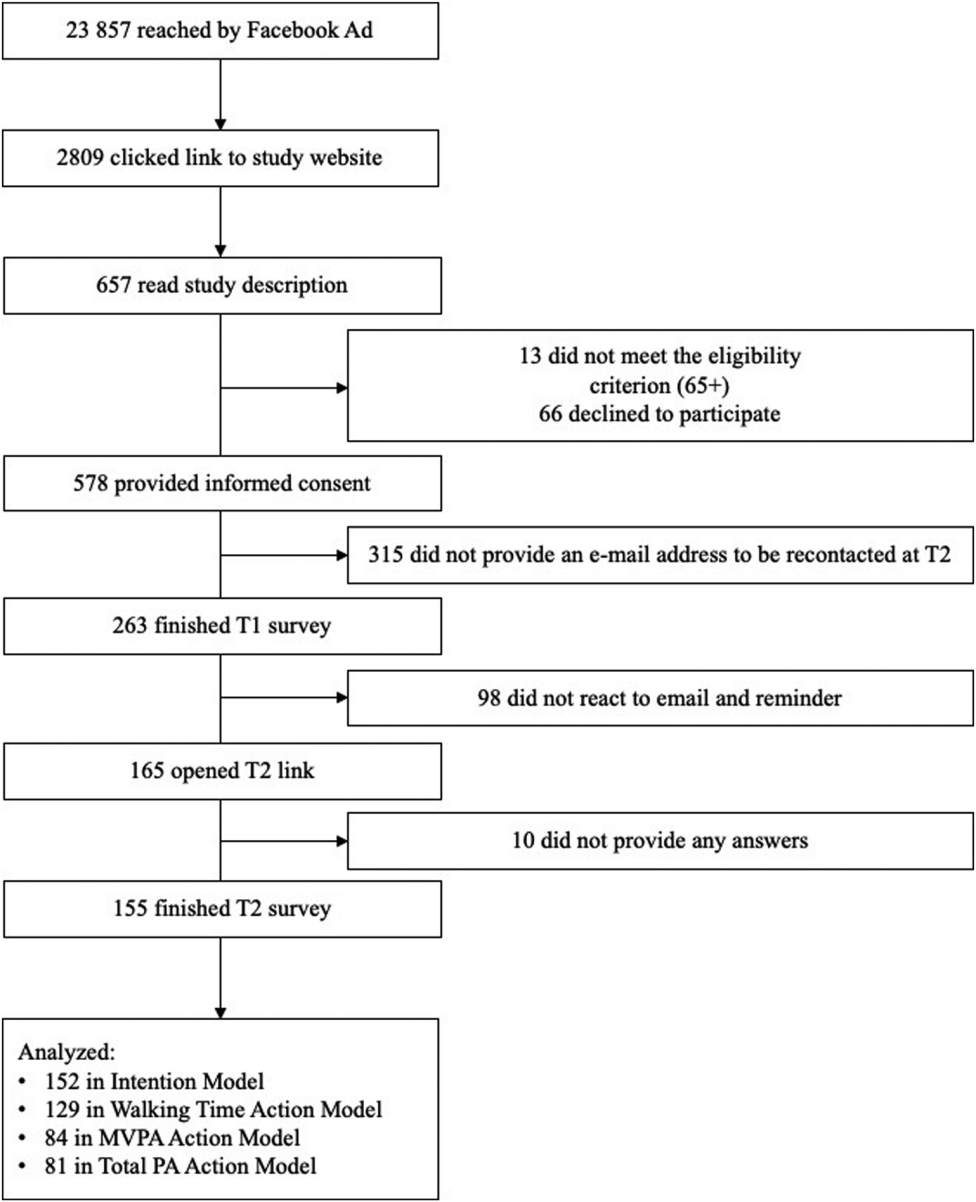
Flowchart of study process. Additionally, the survey was distributed via snowballing (*N* unknown).

No explicit exclusion criteria were set. The survey was then administered online with Qualtrics XM software. The link was distributed via Facebook advertisements, flyers, and forum entries. After providing written informed consent to the study, participants completed an online questionnaire (*N* = 263) where they were asked about their physical activity, questions about the HAPA constructs, and how fearful they were about the pandemic and contracting the virus. One month later, participants (*N* = 155; dropout rate = 40.8%) completed the same questionnaire a second time (see Figure 1). Further information about the study process can be retrieved from the flowchart in Figure 2.

### Measures

#### Physical activity

Physical activity was measured with the International Physical Activity Questionnaire (IPAQ). The IPAQ is a validated questionnaire that measures self‐reported physical activity (Craig et al., 2003). It measures the amount of light physical activity, moderate to vigorous physical activity, and time spent sitting in hours and minutes per week and in days per week.

The minutes of walking per week of each participant was then calculated by transforming the hours of walking into minutes. Moderate to vigorous physical activity (MVPA) was calculated by transforming the hours of vigorous and moderate activity into minutes and then summing both values to arrive at the MVPA score. The total physical activity was calculated by summing the minutes spent walking and the MVPA.

#### HAPA variables

The motivational factors of the HAPA model were measured with three items each. All item answers ranged from 1 (*not at all*) to 5 (*very strong*). The complete list of items used can be found in Table S2 at https://osf.io/j7e4z/?view_only=aebc66fb117748faa4e6b8a8cfdd4c75.

The items for outcome expectancies (α_T1_ = .67; α_T2_ = .60) and motivational self‐efficacy (α_T1_ = .94; α_T2_ = .86) were adapted from Schwarzer (2008), and the items for risk perception (α_T1_ = .83; α_T2_ = .95) were adapted from Bierbauer et al. (2017). Volitional factors were measured by adapting four items from Schwarzer (2008) for intention (α_T1_ = .89; α_T2_ = .86) and three items for action planning (α_T1_ = .93; α_T2_ = .94) and coping planning (α_T1_ = .89; α_T2_ = .90). Action control (α_T1_ = .94; α_T2_ = .92) was adapted from Sniehotta et al. (2006).

#### Fear of Covid‐19

The fear of Covid‐19 was measured with the Swine Flu Inventory (SFI; Wheaton et al., 2012). The SFI was originally used to measure the fear of the H1N1 influenza virus but was modified to fit the current situation by changing its focus to the Covid‐19 pandemic. It was then used to measure the fear of Covid‐19 by inquiring about concerns about the Covid‐19 pandemic, the perceived likelihood of contracting Covid‐19, the perceived severity of infection, avoidance of certain places, the use of safety behaviors, and exposure to information about Covid‐19 (α_T1_ = .68; α_T2_ = .71). Because the scale had a low overall Cronbach's alpha, indicating that not all items represented the fear of Covid as an emotional reaction, an exploratory factor analysis was conducted. Two factors were found: One factor was conceptually closer to the construct of risk perception, which was not in line with the research question, so the second factor better depicted emotional fear and thus was chosen for the analyses (Table S3 containing the factor analysis and Table S4 with the adapted and translated items can be found here: https://osf.io/j7e4z/?view_only=aebc66fb117748faa4e6b8a8cfdd4c75). The Cronbach's alpha for this second factor was α_T1_ = .73 at T1 and α_T2_ = .72 at T2. Moreover, the item “To what extent do you believe that Covid‐19 could become a ‘pandemic’ in Europe?” was not included in the questionnaire because Covid‐19 was already declared a global pandemic on March 11, before the initial distribution of the survey (WHO, 2020). Responses to all items were gathered on Likert scales with four increments ranging from one *least likely* to five *most likely*.

#### Sociodemographic data

Participants were asked about their retirement status (yes; no), age of retirement, and possible employment after retirement (no; yes, and the employment percentage). Information was also gathered about their gender, civil status (single; married; divorced; widowed; in civil union; dissolved union), and highest educational status (primary school; secondary school; apprenticeship; college; technical college; university; other). Other questions concerned their living situation (retirement home; assisted living; alone in an apartment or house; together with a partner in an apartment or house; together with family in an apartment or house), such as where they lived, how many people they lived with, and which people (e.g., spouse) they lived with. Lastly, the survey asked about their socioeconomic status (I do not have enough money to pay my expenses; I have enough money to pay my expenses; I have more than enough money to pay my expenses) and health status (1 = *poor*; 5 = *very good*).

### Data and analysis

First, a dropout analysis was conducted to compare those who completed both surveys (*n* = 152) with those who dropped out after T1 (*n* = 111) with independent t‐tests. In the main analyses, we handled missing data using listwise deletion, because multiple imputation is not recommended when missing data exceeds 40% (Jakobsen et al., 2017). Still, to test the robustness of the results, we conducted sensitivity analyses using data substituted by multiple imputation (Sterne et al., 2009). Because the results did not substantively differ between the two methods, we added these results to the supplementary material (https://osf.io/j7e4z/?view_only=aebc66fb117748faa4e6b8a8cfdd4c75).

In the main analyses, a repeated‐measures analysis of variance (ANOVA) was conducted first to investigate the development of physical activity after the first lockdown. Then, two linear regression analyses were conducted to investigate correlates of physical activity after lockdown. The first model tested the motivational factors at T1 as predictors of the intention to remain physically active at T2. The second model tested the volitional factors at T1 and previous physical activity as predictors of the amount of physical activity at T2. As a sensitivity analysis, models were computed again, adding age, gender, education, and socioeconomic status and health status as covariates. To improve the interpretation of the findings, all independent variables representing the HAPA factors were grand‐mean‐centered by subtracting the sample mean value from the individual value of the participants (e.g., Asparouhov & Muthen, 2006). Outliers were approached to the distribution by replacing them with the highest value, which was still within two standard deviations (SD) of the mean (e.g., Amidan et al., 2005). For better understanding of the results, the effect sizes (f^2^) for each coefficient were calculated and reported (Selya et al., 2012). For the interpretation of these, we reference Cohen (1988), where an f^2^ of .02 represents a small effect, .15 a medium effect, and .35 a large effect.

To test the influence of fear of Covid‐19 on the relationship between intention at T1 and behavior at T2, a moderator analysis was conducted with fear of Covid‐19 at T1 as a continuous moderator. In case of significant moderation, a simple slopes analysis was conducted, testing the intention–behavior relationship at low fear of Covid‐19 (= *M* − 1 *SD*), average fear, and high fear (= *M* + 1 *SD*). All analyses were computed with jamovi version 1.2.27.0 (2020) or SPSS 27, and all supplementary materials are available online (https://osf.io/j7e4z/?view_only=aebc66fb117748faa4e6b8a8cfdd4c75).

## RESULTS

### Participants characteristics

Participants were on average 69.9 years old (*SD* = 4.3). Of these, 68.8% (*n* = 181) were women, 93.2% (*n* 187) were retired (mean retirement age 62.8 years; *SD* = 3.4), and 59.3% (*n* = 156) married. Most had completed an apprenticeship or higher education, and most were in good or very good health (*M* = 4.02; *SD* = 0.9). Some 61.2% (*n* = 161) lived with their partner (Table S1 with all descriptive statistics of the sociodemographic variables can be found at https://osf.io/j7e4z/?view_only=aebc66fb117748faa4e6b8a8cfdd4c75).

### Dropout analysis

Dropouts did not significantly differ from completers in socioeconomic status (*t*(260) = 1.71, *p* = .574), mean walking time at T1 (*t*(229) = 0.30, *p* = .762), mean MVPA at T1 (*t*(161) = 0.35, *p* = .725) and total PA T1 (*t*(159) = 0.44, *p* = .659). However, health status (*t*(259) = −2.44, *p* = .016) and education (*t*(207) = −2.40, *p* = .014) were significantly better among completers. Further, self‐efficacy (*t*(260) = −2.25, *p* = .025), intention (*t*(260) = −3.11, *p* = .002), and coping planning (*t*(249) = −2.89, *p* = .004) at T1 were significantly higher in completers than dropouts (see Table S5 at https://osf.io/j7e4z/?view_only=aebc66fb117748faa4e6b8a8cfdd4c75).

### Physical activity development over time

On average, participants engaged in 147‐min walking time at T1 (*SD* = 7.9) and 141 min at T2 (*SD* = 6.8) at T2. The mean walking time did not change significantly between time points (*F*(1,131) = 0.58, *p* = .446, *η* ^2^_*p*_ = .004). For the MVPA, participants engaged on average in 284 min at T1 (*SD* = 13.6) and 307 min at T2 (*SD* = 17.1). This was similar in the imputed data set (see Figure 2). When only analyzing completers, the mean MVPA did not change significantly between time points (*F*(2,144) = 1.91, *p* = .170, *η* ^2^_*p*_ = .022). However, the same analysis was conducted with the imputed data: The mean MVPA increased significantly from T1 to T2 (*F*(1,262) = 5.05, *p* = .025, *η* ^2^_*p*_ = .019). Lastly, the average total physical in minutes at T1 (*SD* = 19.8) and 460 min at T2 (*SD* = 22.5) and did not change over time (*F*(1,83) = 1.64, *p* = .203, *η* ^2^_*p*_ = .019).

### Predicting intention and physical activity after lockdown

To test whether the motivational factors of the HAPA model at T1 correlate with intention to be active at T2, a linear regression was conducted (see Table 1). The descriptive statistics of all factors (Table S1) and the correlations between them (Table S6) can be found here: https://osf.io/j7e4z/?view_only=aebc66fb117748faa4e6b8a8cfdd4c75.

**TABLE 1:** Linear regression analysis of the intention to be physically active at T2

								95% CI	
		*B*	*SE*	*β*	*T*	*p*	*f* ^2^	LL	UL
1	Intercept	3.99	0.06		64.66	<.001***		3.86	4.11
	Self‐efficacy T1	0.34	0.06	.40	5.54	<.001***	.21	0.22	0.46
	Risk perception T1	0.10	0.05	.12	1.79	.075	.01	−0.01	0.20
	Outcome expectancies T1	0.23	0.07	.24	3.27	.001**	.06	0.09	0.37
2	Intercept	3.17	1.13		2.81	.006*		0.94	5.41
	Self‐efficacy T1	0.26	0.06	.30	4.01	<.001***	.11	0.13	0.38
	Risk perception T1	0.12	0.06	.15	2.12	.036*	.02	0.01	0.22
	Outcome expectancies T1	0.14	0.08	.15	1.87	.064	.02	−0.01	0.29
	Age	0.01	0.01	.02	0.25	.805	−.01	−0.02	0.03
	Gender	−0.08	0.13	−.04	−0.57	.570	<.01	−0.33	0.19
	Health status	0.24	0.08	.24	3.04	.003*	.06	0.09	0.40
	Socioeconomic status	−0.29	0.13	−.16	−2.25	.027*	.04	−0.55	−0.04
	Education	0.05	0.05	.06	0.89	.375	.01	−0.06	0.16

*Note*: *B*, unstandardized regression coefficient; *SE*, standard error; *β*, standardized regression coefficient; all predictors were grand‐mean‐centered,

* *p* < .05.

** *p* < .01.

*** *p* < .001.

Intention to be physically active at T2 was significantly predicted by self‐efficacy and outcome expectancies but not risk perception at T1. The overall model fit was adj. R^2^ = .29.

Only partially in line with the hypothesis, when age, gender, education, socioeconomic status, and health status were added to the model, self‐efficacy remained significant, and risk perception attained significance, but outcome expectancies were no longer significant. The overall model fit was adj. R^2^ = .35.

To test the association of volitional HAPA factors at T1 on walking time, MVPA and total PA at T2, linear regressions were conducted (see Table 2). When the model was analyzed for the walking time (*n* = 129), contrary to hypotheses, the self‐reported walking time at T2 was not significantly predicted by any volitional factor at T1. The overall model fit was adj. R^2^ = −.02. When the walking time at T1 was added as covariate, none of the HAPA variables attained significance, but the walking time at T1 predicted the walking time at T2. The overall model fit was adj. R^2^ = .111. When adding the covariates, only the walking time at T1 remained significant.

**TABLE 2:** Linear regression analysis of physical activity at T2

		Walking time								MVPA								Total PA							
								95% CI								95% CI								95% CI	
		*B*	*SE*	*ß*	*T*	*p*	*f* ^2^	LL	UL	*B*	*SE*	*ß*	*T*	*p*	*f* ^2^	LL	UL	*B*	*SE*	*ß*	*T*	*p*	*f* ^2^	LL	UL
1	Intercept	139.93	7.47		18.74	<.00***		125.15	154.71	297.57	19.34	0	15.39	<.001***		259.07	336.06	449.25	26.25		15.39	<.001***		259.07	336.06
	Intention T1	−0.55	13.7	−.01	−0.04	.968	0	−27.67	26.57	−16.2	37.75	−.08	−0.43	.669	0	−91.33	58.94	−16.2	37.75	−.08	−0.43	.669	0	−91.33	58.94
	Action planning T1	10.15	9.64	.13	1.05	.294	.01	−8.92	29.22	−2.74	24.42	−.02	−0.11	.911	<.01	−51.35	45.87	−2.74	24.42	−.02	−0.11	.911	<.01	−51.35	45.87
	Coping planning T1	−3.26	11.79	−.04	−0.28	.783	<.01	−26.61	20.08	18.3	31.1	.12	0.59	.558	.01	−43.61	80.2	18.3	31.1	.12	0.59	.558	−.09	−43.61	80.2
	Action control T1	0.32	10.4	.01	0.03	.976	0	−20.27	20.9	19.13	26.43	.14	0.72	.471	.01	−33.47	71.74	19.13	26.43	.14	0.72	.471	0	−33.47	71.74
2	Intercept	90.57	12.87	0	7.04	<.001***		65.11	116.04	168.36	40.44		4.16	<.001***		87.84	248.88	168.36	40.44		4.16	<.001***		87.84	248.88
	Intention T1	−2.27	12.73	−.02	−0.18	.859	.03	−27.47	22.93	−9.4	35.27	−.05	−0.27	.790	0	−79.62	60.82	−9.4	35.27	−.05	−0.27	.790	0	−79.62	60.82
	Action planning T1	15.19	9.02	.20	1.68	.095	.02	−2.66	33.04	−12.92	22.96	−.08	−0.56	.575	<.01	−58.64	32.79	−12.92	22.96	−.08	−0.56	.575	<.01	−58.64	32.79
	Coping planning T1	−9.48	11.04	−.12	−0.86	.392	.01	−31.34	12.37	20.17	29.02	.13	0.69	.489	.01	−37.62	77.95	20.17	29.02	.13	0.69	.489	0	−37.62	77.95
	Action control T1	−1.58	9.67	−.02	−0.16	.871	0	−20.72	17.56	15.44	24.68	.11	0.63	.534	0	−33.7	64.57	15.44	24.68	.11	0.63	.534	0	−33.7	64.57
	Physical activity T1	0.34	0.07	.39	4.55	<.001***	.17	0.19	0.49	0.47	0.13	.37	3.57	.001**	.13	0.21	0.74	0.47	0.13	.37	3.57	.001**	.23	0.21	0.74
3	Intercept	−103.48	121.76		−0.85	.397		−344.61	137.65	179.88	304.57		0.59	.557		−427.13	786.89	179.88	304.57		0.59	.557		−427.13	786.89
	Intention T1	−1.4	13.41	−.01	−0.1	.917	0	−27.94	25.15	−1.21	36.38	−.01	−0.03	.974	0	−73.72	71.3	−1.21	36.38	−.01	−0.03	.974	<.01	−73.72	71.3
	Action planning T1	13.67	9.1	.18	1.5	.136	.02	−4.36	31.69	−13.7	23.59	−.09	−0.58	.563	<.01	−60.71	33.31	−13.7	23.59	−.09	−0.58	.563	0	−60.71	33.31
	Coping planning T1	−7.08	11.37	−.09	−0.62	.535	<.01	−29.61	15.44	21.27	30.18	.14	0.7	.483	.01	−38.89	81.43	21.27	30.18	.14	0.7	.483	0	−38.89	81.43
	Action control T1	−4.28	10.09	−.06	−0.42	.672	<.01	−24.26	15.69	16.56	25.19	.12	0.66	.513	0	−33.64	66.77	16.56	25.19	.12	0.66	.513	0	−33.64	66.77
	Physical activity T1	0.33	0.08	.38	4.44	<.001***	.18	0.18	0.48	0.48	0.14	.38	3.5	.001**	.12	0.21	0.75	0.48	0.14	.38	3.5	.001**	.25	0.21	0.75
	Age	2.02	1.55	.11	1.31	.194	.01	−1.04	5.08	−1.24	3.56	−.04	−0.35	.728	−.07	−8.33	5.85	−1.24	3.56	−.04	−0.35	.728	−.03	−8.33	5.85
	Gender	−13.43	14.71	−.08	−0.91	.363	.01	−42.56	15.7	−35.79	35.44	−.12	−1.01	.316	.01	−106.42	34.84	−35.79	35.44	−.12	−1.01	.316	.02	−106.42	34.84
	Socioeconomic status	18.07	14.78	.11	1.22	.224	.01	−11.21	47.35	46.18	36.33	.14	1.27	.208	.02	−26.24	118.59	46.18	36.33	.14	1.27	.208	.03	−26.24	118.59
	Health status	7.02	8.81	.07	0.8	.427	<.01	−10.43	24.47	−2.68	22.88	−.01	−0.12	.907	0	−48.27	42.92	−2.68	22.88	−.01	−0.12	.907	0	−48.27	42.92
	Education	2	6.01	.03	0.33	.739	0	−9.89	13.9	9.78	15.37	.08	0.64	.526	.01	−20.84	40.41	9.78	15.37	.08	0.64	.526	<.01	−20.84	40.41

*Note*: *B*, unstandardized regression coefficient; *SE*, standard error; *β*, standardized regression coefficient; all predictors were grand‐mean‐centered.

* *p* < .05.

** *p* < .01.

*** *p* < .001.

The results of the linear regression for MVPA (*n* = 84) were similar. Only the MVPA at T1 predicted the MVPA at T2. The first model had a fit of adj. R^2^ = −.02, when adding the MVPA at T1 the fit changed to adj. R^2^ = .12, and lastly the fit when adding all covariates was adj. R^2^ = .09.

Lastly, similar results were also found for the total amount of physical activity (*n* = 81). Only the past total physical activity could predict the total amount of physical activity at T2. The first model with only the volitional factors had a fit of adj. R^2^ = −.04, when adding the total amount of physical activity at T1 the fit changed to adj. R^2^ = .16, and lastly the fit when adding all covariates was adj. R^2^ = .14.

### Sensitivity analyses

After multiple imputation, the intention to be physically active at T2 was significantly predicted by self‐efficacy and outcome expectancies but not risk perception at T1. The range of the model fit over all five imputed data sets varied from adj. R^2^ = .11 to adj. R^2^ = .16. Only partially in line with the hypothesis, when the covariates were added to the model, self‐efficacy remained significant but risk perception and outcome expectancies were no longer significant predictors of intention. The model fit ranged from adjusted R^2^ = .12 to adj. R^2^ = .17 over all imputations. The results of the action model for walking time, MVPA, and total PA were unchanged after imputation (see all sensitivity analyses with the imputed datasets in Supplement 7 and 8: https://osf.io/j7e4z/?view_only=aebc66fb117748faa4e6b8a8cfdd4c75).

### Does fear of Covid‐19 moderate the intention–behavior relationship?

Contrary to our hypothesis, the effect of intention on walking time (*B* = −17.81; CI [−36.99, 1.36], *p* = .069), MVPA (*B* = −20.2; CI [−64.27, 23.90], *p* = .369) or total physical activity (*B* = −33.1; CI [−91.11, 25.00], *p* = .265) was not moderated by fear of Covid‐19 either for completers or when including imputed data (see Supplement 9: https://osf.io/j7e4z/?view_only=aebc66fb117748faa4e6b8a8cfdd4c75).

### Exploratory analyses

However, because walking time, MVPA, and the total amount of physical activity at T1 were the only factors that significantly predicted MVPA at T2, an exploratory moderation analysis was conducted with these variables and the fear of Covid‐19. No moderator effect was found for the waking time (*B* = −0.049, CI [0.10, −0.49], *p* = .628).

For MVPA, the fear of Covid‐19 moderated the effect of MVPA at T1 and physical activity at T2 (*B* = −0.34, CI [−0.33, −0.04], *p* = .001). A simple slopes analysis revealed that MVPA at T1 was only predicted by MVPA at T2 for older adults with low (1 SD below the mean; *B* = 0.77, *SE* = 0.16, CI [0. 451, 1.09], *p* = <.001) to average fear of Covid‐19 (*B* = 0.53, *SE* = 0.12, CI [0. 29, 0.76], *p* = <.001). For older adults with high fear, the effect was disrupted (1 SD below the mean of Covid fear; *B* = 0.28, *SE* = 0.15, CI [−0.015, 0.58], *p* = .063). Similar results were found for the imputed data set. However, this effect disappeared after identifying and removing potential bivariate outliers in the completers (*B* = −0.09, CI [−0.35, 0.17], *p* = .500) and imputed data set (*B* = −0.09, CI [−0.22, 0.02], *p* = .113). Moderation analysis for the total amount of physical activity was again not significant (*B* = −0.194, CI [−0.425, −0.036], *p* = .098). The results remained substantially unchanged when the imputed data was included (for all results, see Supplement 10: https://osf.io/j7e4z/?view_only=aebc66fb117748faa4e6b8a8cfdd4c75).

## DISCUSSION

The purpose of this study was to gain a better understanding of whether and why some older adults' physically activity changed after the lockdown of the first wave of the Covid‐19 pandemic. The results showed that physical activity stayed mainly the same or improved over time as lockdown restrictions were lifted. Partially in line with our hypotheses, self‐efficacy and health status robustly positively predicted the intention to be physically active. Thus, older adults with higher self‐efficacy showed stronger intentions to be active as lockdown restrictions eased. Contrary to our hypothesis, none of the volitional factors of the HAPA model predicted physical activity after lockdown. Only past physical activity predicted activity after lockdown. Our results indicated that the fear of Covid‐19 did not qualify the intention–behavior relationship. Exploratory results provided some evidence that fear of Covid‐19 can moderate the past behavior–future behavior relationship such that past behavior might not be predictive of future behavior in fearful individuals. However, these results are preliminary as they did not hold when outliers were removed.

### Physical activity of older adults after the lockdown

As lockdown restrictions eased, physical activity over time was consistent or improved further. This is encouraging given the evidence that physical activity was negatively impacted by the restrictions faced in the first wave of Covid‐19 (Naughton et al., 2020), especially among older adults (Bu et al., 2021; Carriedo et al., 2020).

Two motivational factors of the HAPA model, self‐efficacy and outcome expectancies, related to the intention to be physically active. The results on risk perception were inconclusive as this effect was not significant when analyzing the completers. The nonsignificance of risk perception is a common result and was mentioned, for example, in Zhang et al.'s (2019) meta‐analysis, which concluded that the effects of outcome expectancies and risk perception were small and that self‐efficacy was the most promising factor in predicting health behaviors in general. Similar findings were observed in a study by Bierbauer et al. (2017), who found that risk perception had no significant association with older adults' intention to be physically active and who argued that the perception of being at risk is not equally important to all health behaviors.

Outcome expectancies related positively to intention, which is in line with previous findings (Williams et al., 2005), where outcome expectancy was found to be a central construct in social‐cognitive models of physical activity. However, some evidence shows that health‐related outcome expectancy has no effect on intentions or behavior, especially in older adults (Gellert et al., 2012). This uncertainty is reflected in our regression results, which are not as robust as those for self‐efficacy. Outcome expectancies lost their significance when the covariates were added.

The evidence on the self‐efficacy effect is well funded in social cognitive theory (Bandura, 1998) and robust in our analyses. Self‐efficacious individuals approach difficult tasks as challenges to be mastered rather than as threats to be avoided (Bandura & Ramachaudran, 1994). This could explain why self‐efficacy is so important during the global crisis of the Covid‐19 pandemic, because self‐efficacious individuals would be more likely to view remaining active as a challenge to be tackled than a situation that would overwhelm them, which is in line with research that shows that overall people reported significantly less benefit, less enjoyment, and less confidence to remain physically active during the Covid‐19‐pandemic (Lesser & Nienhuis, 2020) making it plausible that self‐efficacy could help maintain a strong intention to be active nevertheless. Moreover, self‐efficacy was also found to predict the intention to perform pandemic‐specific preventive behaviors like the intention to perform social distancing (Hamilton et al., 2020) and handwashing in older adults particularly (Duan et al., 2022). Thus, self‐efficacy seems an important resource and protective factor for many health‐relevant behaviors of older adults during the pandemic.

This notion of personal resources could also explain the interesting relationship between health status and the intention to remain active. Healthier individuals could be less absorbed with the pandemic's impact on the health system and its consequences for their treatment (e.g., Wosik et al., 2020) and therefore have more resources for being active than individuals who are in poorer health.

Volitional factors alone failed to predict differences in physical activity after lockdown. This is further highlighted by the low or even negative R^2^ values in the action model, indicating that volitional factors are not as important for the recovery of physical activity. Therefore, these variables cannot be considered reliable predictors of the dependent variable (e.g., Chicco et al., 2021). This stands in contrast to Lin et al.'s (2020) and Zhang et al.'s (2020) finding that volitional factors such as especially coping and action planning significantly predict health behaviors specific to Covid‐19, such as washing hands. Ziegelmann et al. (2006) also found that more detailed action plans led to more physical activity in older adults up to 6 months after the end of an intervention. And Wolff et al. (2016) confirm that formulating coping and action plans leads to more physical activity in an intervention. Thus, our findings about action and coping planning contrast with previous research overall. However, some previous evidence has shown that action planning is not always useful for older adults, especially for physical activity (Warner et al., 2016).

Moreover, intention did not predict behavior, which is in contrast to numerous findings and theories that assume that the intention to perform a certain health behavior is a key predictor of that behavior (Sheeran, 2002). Our results could be an indicator of the intention–behavior gap (Sheeran & Webb, 2016). However, because the mean physical activity in our sample was constantly higher than official recommendations, it seems unlikely that our results are due to the intention–behavior gap. An alternative explanation is that the lack of relationship between intention and behavior is due to strong automatization of the behavior: A physical activity habit (Hagger, 2019). Sheeran and Webb (2016), for example, showed that the predictive value of intention on behavior declines with greater experience, which reflects increased automatization of the behavior. This explanation is supported by a meta‐analysis that showed that the intention–behavior gap is smaller in older adults, being experienced than in younger adults (Hagger et al., 2002). This explanation is further supported by our observation that the amount of physical activity at T1 was significantly related to the amount of activity at T2. This is further underlined by Hagger et al. (2018), who state that past behavior typically exhibits larger effects on future behavior than other social‐cognitive factors such as intention due to implicit, unconscious processes. Moreover, Di Maio et al. (2021) found that the intention–activity relationship was moderated by habit strength, suggesting that habit has a compensatory effect. And in light of the pandemic, preventive behaviors like social distancing were also strongly predicted by habit, suggesting that it could be also central for the maintenance of other behaviors during times when it is more difficult to exhibit a certain behavior (Hagger et al., 2021). Also, other studies already show that habit mediates the relationship between past and current physical activity in general in older adults (van Bree et al., 2015). Therefore, when a behaviour becomes habitual, like in this case physical activity, volitional factors become less important. Instead, habit plays a greater role (e.g., Rhodes & De Bruijn, 2010). However, certain conditions can cause disruption to routines and restrictions on personal lives during a pandemic, as postulated by Spence et al. (2021), and hence moderate this effect.

### Moderator effects of fear of Covid‐19

Contrary to our hypotheses, the relation between intention and behavior was not moderated by fear of Covid‐19. However, an exploratory analysis showed some evidence that fear of Covid‐19 can disrupt the relation between past and current behavior. This suggests that experiencing fear may disrupt a habitual behavior. This is in line with habit theory, which states that changing context can cause habit to discontinue. Interestingly, this was only found for MVPA, and not for walking time or total PA. Perhaps, activities like walking are less impacted by restrictions such as those imposed by lockdown. The effect for MVPA is preliminary, because the finding was not robust when bivariate outliers were removed. This can be due to the small sample size because the influence of outliers increases the smaller the sample (Van Selst & Jolicoeur, 1994). However, this result can be seen as hypothesis‐generating, warranting future research.

#### Limitations and further directions

Overall, the sample depicts a healthy and active population of older adults. The oversampling of healthy and active participants may be due to a selection bias of active and interested individuals caused by the use of social media ads as primary recruitment tool. Studies, for example, have shown that smartphone expertise, an inclusion criterion in our study, can correlate positively with health outcomes (Mohlman & Basch, 2021). The high panel attrition may have further contributed to the selection bias. Our dropout analysis indicated that healthier, more educated participants with a higher intention to be active were also more likely to complete both time points. Therefore, the study results may not be generalizable to the entire population of older adults. Further, due to the rapid onset of the pandemic, we did not obtain data before the lockdown. Therefore, we cannot be sure that our sample's physical activity decreased during the lockdown, even though this seems likely given evidence from other studies (Naughton et al., 2020). Encouragingly, our analyses with completers and the imputed data largely converged, indicating the robustness of our findings.

Despite these limitations, the present study has enhanced the understanding of older adults' physical activity after lockdown. Overall, our findings align well with those from other studies in that healthy older adults living at home may be less severely affected by the pandemic than previously assumed (Knepple Carney et al., 2021). Further, the findings support research showing that self‐efficacy has an important influence on the intention to be active when staying active is difficult and less enjoyable, making it potentially an important factor to target in behavior maintenance interventions during pandemic times. Especially because it is also linked to other pandemic‐specific health behavior like handwashing (Zhang et al., 2020) and mask use (Duan et al., 2022) making it an important protective factor for health relevant behaviors during Covid‐19. Finally, strong habits may be a protective factor for maintenance of physical activity during the pandemic, making their promotion even more important. The preliminary finding that fear could potentially disrupt this habitual relationship in older adults provides an interesting avenue for further investigating moderators of the maintenance of healthy habits during a pandemic.

## CONFLICTS OF INTEREST

The authors declared that they had no conflicts to disclose.

## ETHICS STATEMENT

The study was approved by the Ethics Committee of the University of Bern.

## Data Availability

The data are available on request from the first author.
